# GABAergic Mechanisms Can Redress the Tilted Balance between Excitation and Inhibition in Damaged Spinal Networks

**DOI:** 10.1007/s12035-021-02370-5

**Published:** 2021-04-07

**Authors:** Graciela Lujan Mazzone, Atiyeh Mohammadshirazi, Jorge Benjamin Aquino, Andrea Nistri, Giuliano Taccola

**Affiliations:** 1 Instituto de Medicina Traslacional (IIMT), CONICET-Universidad Austral, Av. Juan Domingo Perón 1500, B1629AHJ, Pilar, Argentina; 2 Neuroscience Department, International School for Advanced Studies (SISSA), Via Bonomea 265, 34136 Trieste, Italy

**Keywords:** GABA, Spinal circuits, Spinal cord injury, Spinal shock, Neuroprotection, Spasticity

## Abstract

Correct operation of neuronal networks depends on the interplay between synaptic excitation and inhibition processes leading to a dynamic state termed balanced network. In the spinal cord, balanced network activity is fundamental for the expression of locomotor patterns necessary for rhythmic activation of limb extensor and flexor muscles. After spinal cord lesion, paralysis ensues often followed by spasticity. These conditions imply that, below the damaged site, the state of balanced networks has been disrupted and that restoration might be attempted by modulating the excitability of sublesional spinal neurons. Because of the widespread expression of inhibitory GABAergic neurons in the spinal cord, their role in the early and late phases of spinal cord injury deserves full attention. Thus, an early surge in extracellular GABA might be involved in the onset of spinal shock while a relative deficit of GABAergic mechanisms may be a contributor to spasticity. We discuss the role of GABA A receptors at synaptic and extrasynaptic level to modulate network excitability and to offer a pharmacological target for symptom control. In particular, it is proposed that activation of GABA A receptors with synthetic GABA agonists may downregulate motoneuron hyperexcitability (due to enhanced persistent ionic currents) and, therefore, diminish spasticity. This approach might constitute a complementary strategy to regulate network excitability after injury so that reconstruction of damaged spinal networks with new materials or cell transplants might proceed more successfully.

## Synaptic Inhibition Is an Important Component of Spinal Locomotor Networks

**Fig. 1:**
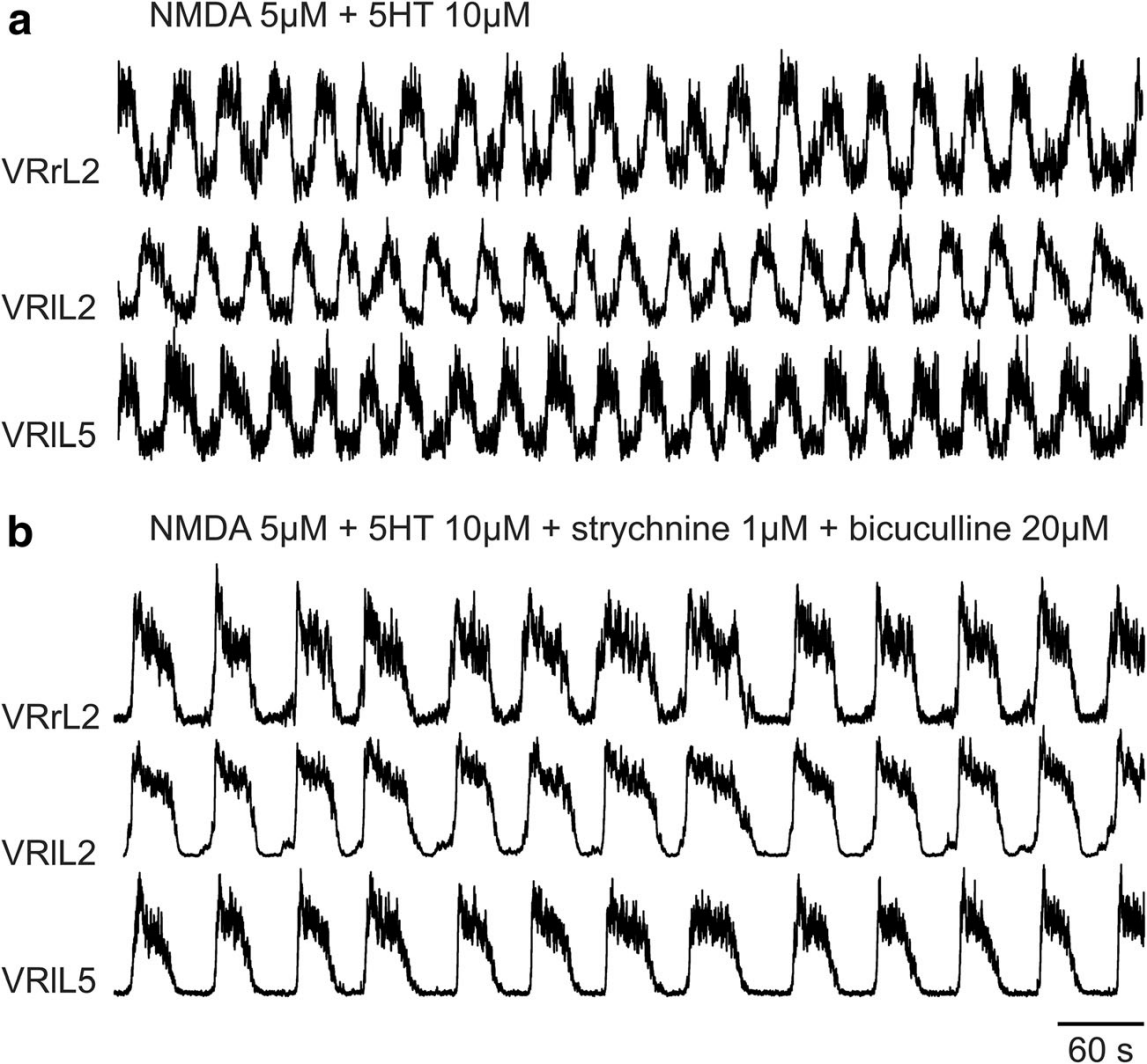
During locomotor patterns, fast synaptic transmission is essential to allow the sequential activation of antagonistic motor pools innervating flexor and extensor hindlimb muscles. **a** A stable locomotor-like rhythm is induced in the spinal cord isolated from a neonatal rat by co-application of the glutamate agonist NMDA plus 5HT. The rhythm reflects the basic pattern of activation of lower limb muscles during real locomotion, which is composed of electrical discharges characteristically alternating between right (r) and left (l) ventral roots (VRs, exemplified in this figure at the second lumbar segment; L2) and between flexor (L2)- and extensor (L5)-related ventral roots on the same side of the cord (shown in this figure as the left L2 and L5). **b** On the same preparation, strychnine plus bicuculline are further applied to block glycinergic and GABAergic fast inhibitory transmission, respectively. Starting from 30 s after drug application, the double alternating pattern is replaced by a stable and slower rhythm that becomes synchronous among all ventral roots (unpublished traces, replicating results originally reported by Beato and Nistri, [12])

In mammals, rhythmic motor tasks such as locomotion require balanced network activity based on the coordinated interaction between synaptic excitation and inhibition [1–3]. While inhibition typically dampens neuronal excitability, its overall impact traditionally depends on the reciprocal coupling to excitation in a “push-pull fashion,” whereby inhibition declines as excitation rises and neuron excitability grows, and vice versa [4]. Studies of spinal networks have, however, indicated that, in certain circuits impinging upon motoneurons, synaptic inhibition remains operative even during excitation, suggesting that there are multiple sources of inhibitory inputs beyond the mutual interaction between excitatory and inhibitory local circuits [3]. These observations support the concept of recurrent connectivity [5] that should include a robust component of recurrent inhibition to prevent network instability and ensure multifunction flexibility [6]. In this framework, an important role is played by the neurotransmitter gamma-aminobutyric acid (GABA) that controls not only locomotor cycles but also network assembly during early development [7]. These properties are particularly expressed by a spinal circuit termed central pattern generator (CPG; [8, 9] that can produce rhythmic locomotor activity independent from sensory inputs). Such a process is readily replicated with a model system like the isolated rodent spinal cord which generates alternating rhythmic patterns termed fictive locomotion because of the absence of muscle targets [10]. While excitation is primarily mediated by glutamate and its pharmacological block arrests locomotion [11], blocking inhibition evoked by amino acid transmitters like GABA and glycine suppresses alternation of motor output by the CPG and replaces it with slow rhythmic motor discharges detected synchronously in ventral roots. This phenomenon is exemplified in Fig. 1 in which the fictive locomotor patterns elicited by co-applied *N*-methyl-d-aspartate (NMDA) and serotonin (5HT, see Fig. 1a) and recorded from ventral roots (VRs) are converted into slow synchronous discharges (Fig. 1b).

It should be noted that the GABA receptor antagonist bicuculline [13] is selectively blocking a distinct class of GABA receptors termed GABA A receptors (GABA ARs) known to mediate fast synaptic inhibition [14, 15] as well as to modulate neuronal excitability through extrasynaptic GABA receptors [16, 17]. The term “fast” inhibition, therefore, refers to the short time course underlining the loss of excitability mainly caused by hyperpolarization of the neuronal membrane (for less than 100 ms; [18]). Data in Fig. 1b also indicate that strychnine, a potent glycine receptor antagonist, contributes to block fast inhibition and suggests that, in addition to GABA, glycine is an important mediator of locomotor activity [19, 20].

Indeed, intrasegmental GABAergic and glycinergic interneurons with short axons have been found in ventral laminae where locomotor circuits are located [21]. On in vitro spinal networks, application of strychnine alone evokes irregular and asynchronous discharges while application of bicuculline per se produces a more structured repetitive activity [22, 23]. It may also be suggested that when one type of synaptic inhibition is blocked, the other one can at least in part expand its role because the circuitry is not arrested in a state of sustained excitation. It is noteworthy that the persistent rhythmic activity evoked by the convulsants strychnine and bicuculline is not associated with extensive neuronal or glial death [24, 25], indicating that spinal networks are far more resistant than brain networks to seizure-evoked neurodegeneration [26].

## Principal Properties of GABAergic Mechanisms in the Spinal Cord

GABA is produced by decarboxylation of l-glutamate by glutamic acid decarboxylase (GAD), of which two isoforms exist: the transiently activated GAD65, which synthesizes vesicular GABA to be released by exocytosis, and the constitutively active GAD67, responsible for cytosolic GABA released by paracrine diffusion [27, 28]. In the spinal cord, GAD67 immunostaining has been found in cell bodies and fibers, while GAD65 is mainly located at synaptic terminals [29]. In addition to GABA locally released by spinal neurons and glial cells, GABAergic descending projections from the ventromedial medulla of the brainstem reach ventral and dorsal horns [30–32]. The development of the spinal GABAergic system is guided by several descending projections and the perinatal interruption of these projections impairs the regulation of GABA synthesizing enzymes [33] and receptors in the spinal cord [34]. For instance, interruption of descending serotoninergic input disrupts maturation of spinal GABAergic systems [34].

GABA acts on multiple ionotropic receptors, namely the A subtype, which drives a fast synaptic inhibition and the C subtype, whose role in the spinal cord is however limited, even if functionally expressed in the postnatal mammalian spinal cord [35]. Moreover, GABA acts as mediator of presynaptic inhibition by activating the G protein–coupled B receptor involved in a slower neuromodulating action particularly at presynaptic level via inhibition of calcium conductances [15, 18].

**Fig. 2:**
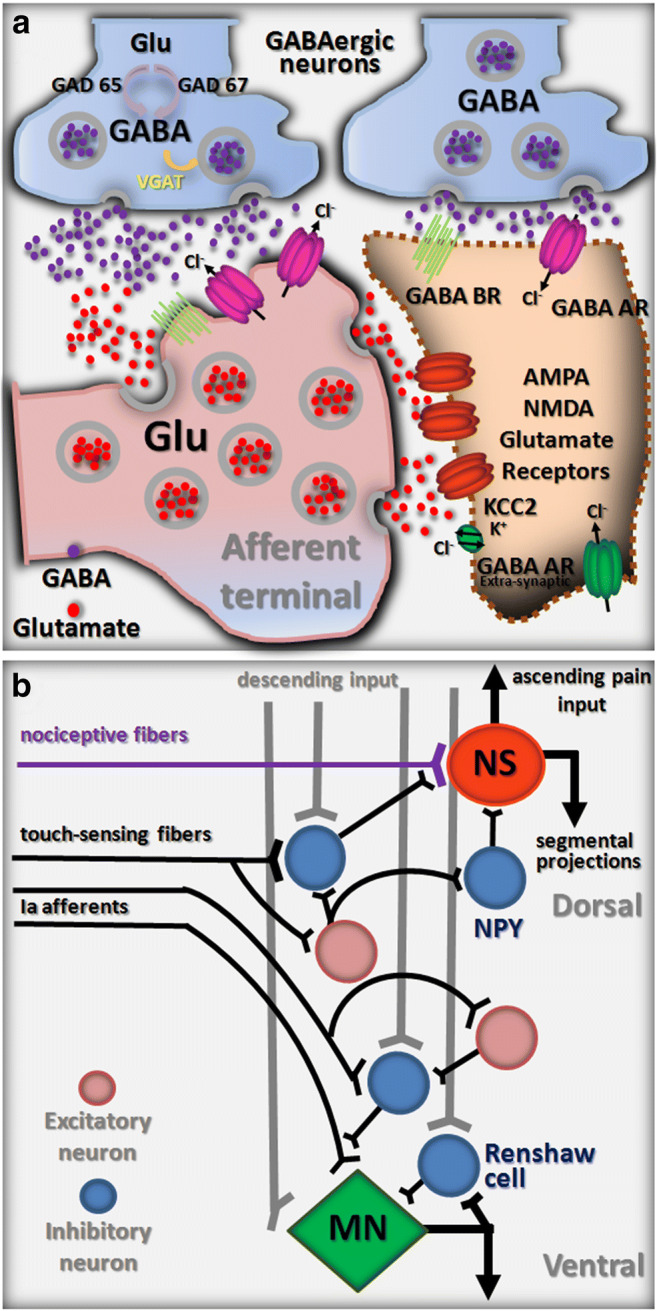
GABA-mediated inhibition at the cellular and network levels. **a** Schematic representation of two prototypical GABAergic synapses mediating pre (left)- and post (right)-synaptic inhibition, respectively. The main cellular and molecular players relevant to a spinal cord injury are depicted as discussed in this review. **b** Simplified wiring diagram of the basic GABAergic circuits involved in presynaptic inhibition of afferent input. NS, nociceptive-specific projection neuron; MN, motoneuron

In adult neurons, GABA A receptor–mediated inhibition is due to the permeation of Cl^-^ (and HCO_3_^-^) through an intrinsic channel that drives an influx of Cl^-^ into the postsynaptic cell (Fig. 2a) [36, 37]. Conversely, in the first postnatal days of life, the opening of GABA ARs coincides with the Cl^-^ electrochemical gradient (driving force) set at the less negative value and, thus, it drives Cl^-^ efflux across the neuronal membrane. This phenomenon decreases intracellular negative charges with consequent cell depolarization from resting potential. It should also be noted that the opening of Cl^-^ channels reduces membrane resistance and temporarily determines a conductance short-circuit (shunting), which limits further depolarization by incoming excitatory inputs. Thus, GABA-mediated depolarization exerts an inhibitory function in neonatal spinal neurons [37]. An action similar to GABA on neonatal neurons is displayed by afferent terminals throughout their maturation and adult stages, due to the high concentrations of intracellular Cl^-^ in Dorsal root ganglions (DRGs) [38].

It is important to emphasize that, in the neonatal spinal cord, the functional outcome of GABA-mediated activity may depend on the location of GABAergic synapses on postsynaptic neurons and their Cl^-^ equilibrium potential [39] because the shunting effect is briefer than the membrane depolarization that, if prolonged, may facilitate excitation [39].

**Fig. 3:**
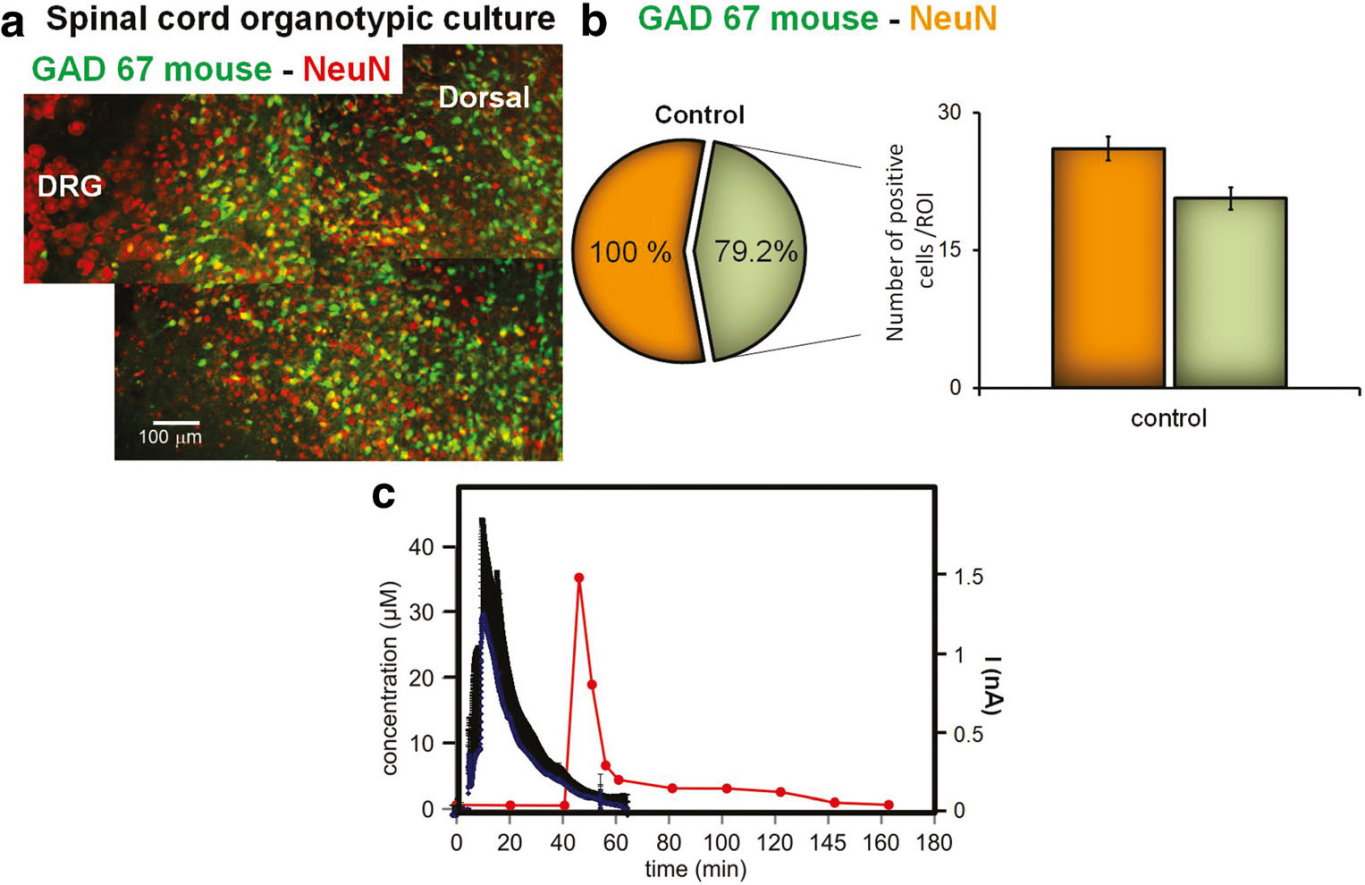
Expression of GABAergic neurons in the spinal cord and real-time glutamate release from spinal cord following experimental spinal cord injury (SCI). **a** Typical neuronal staining with neuronal nuclear protein (NeuN; red) restricted to the spinal cord tissue region in a spinal cord slice of a GAD67-glial filament protein (GFP) expressing mouse (green). Example of 22 DIV slice with two regions of interest (ROIs), namely a dorsal and a ventral horn, and a dorsal root ganglion (DRG). **b** Histograms showing the number of GAD67-positive cells (light green columns) or NeuN-positive cells (orange columns) at 22DIV, in control slices. Inset with the circle chart showing the percentage of GAD67 from NeuN-positive cells (redrawn from Mazzone and Nistri, 2019). **c** Examples of the time-course of endogenous glutamate release detected by glutamate biosensor in cultures that were treated with 0.5 mM kainate (blue traces, mean ± SD, *n*=5 slices). Glutamate concentrations in microdialysis samples collected after spinal cord injury, filled circles (redrawn from [42])

Noteworthily, there is also a subpopulation of extrasynaptic GABA ARs with distinct subunit composition and high affinity to GABA [16, 17, 40], generating tonic modulation of sensory transmission [41]. As exemplified in Fig. 3a, b, for the strong distribution of GABAergic GAD67 neurons in the dorsal horn, the corresponding expression of GABA ARs is intense in inner dorsal laminae (II, III), around the central canal (X), and the ventral horns (VII-IX), where GABA ARs are found at axo-axonic contacts and extra-synaptic sites [43, 44].

## Neuronal Chloride Homeostasis in the Spinal Cord is Regulated by Two Transporters

The synaptic action of GABA and glycine depends on the intracellular concentration of Cl^-^ that is primarily maintained by cation-chloride co-transporters [45]. Among the most important families of Cl^-^ transporters, the Na^+^-K^+^-2Cl^−^ cotransporter 1 (NKCC1) and KCC2 reciprocally control the intracellular Cl^-^ concentration whose efflux causes, for instance, primary afferent-mediated depolarization with depression of excitatory inputs [46, 47]. Cl^-^ transport into the cell is mostly due to NKCC1 activity, whereas KCC2 extrudes Cl^-^ via a fast and concentration-dependent process generated by Na^+^/K^+^-ATPase [46, 47]. Previous studies have demonstrated that Cl^-^ transporter expression and Cl^-^ homeostasis are regulated by developmental changes that include gene transcription modification, posttranslational and trafficking alterations [47–49]. NKCC1 expression is widespread in neurons, glial, blood vessels, and other epithelial cells in the developing and mature central nervous system [50]. On the contrary, KCC2 is restricted to the somatodendritic membrane of mature central neurons and is almost absent in neuronal axons, peripheral neurons, and non-neuronal cells [51, 52]. Due to the broad NKCC1 distribution, NKCC1 null mice have been used to examine the transporter expression and its impact to induce abnormal GABA responses by DRG [53] and cortical neurons [54].

The strength of postsynaptic inhibition, related to Cl^-^ homeostasis, is hampered in several pathophysiological conditions [55] such as seizure, epilepsy, stroke, and ischemic injury [33, 56] and proprioception disorders [57]. Indeed, impaired excitation/inhibition balance due to changed NKCC1 or KCC2 expression was also related to chronic stress [58], brain or peripheral injury [47, 59], and locomotor activity after spinal cord injury [60, 61] or developmental changes [62–64].

In rodent models of spinal cord injury, the role of intracellular chloride concentration and the modulation of cation chloride co-transporter expression have been amply investigated [65, 66]. In particular, synaptic inhibition, KCC2 and NKCC1 expression, and functional recovery were reportedly improved by programmed exercise or bumetanide, a pharmacological antagonist of NKCC1, 28 days after spinal cord transection in rats [67]. Similarly, a reduction in tissue damage and edema was observed by using bumetanide in a spinal cord contusion model [68]. A recent study has shown that the application of anodal trans-spinal direct current stimulation plus bumetanide administration downregulated the expression of NKCC1 after spinal cord contusion with significant amelioration of spasticity and locomotor muscle tone [69]. This is strong evidence that modulation of chloride homeostasis by NKCC1 pharmacological regulation during pathological conditions such as spinal cord injury can favor locomotor network improvement.

## Presynaptic GABAergic Inhibition and Neuropathic Pain

Depolarizing axo-axonic synapses on primary afferent fibers filter incoming input from the periphery via membrane shunting and Na^+^ channel inactivation [38]. This basic wiring scheme fulfills multiple functions in sensory-motor networks. Indeed, a first mechanism to gate pain signals is represented by touch-sensing fibers depolarizing nociceptive primary afferents, thus causing pre-synaptic inhibition of nociceptive input. Furthermore, presynaptic primary afferent depolarization also contributes to shaping motor reflexes and efficiently modulates rhythmic motor behaviors, such as stepping and scratching, in response to proprioceptive input about joint position. Descending commands targeted to local interneurons control the efficiency of presynaptic inhibition triggered by peripheral inputs (Fig. 2b). Although this key frame is complicated by additional neuronal elements that release several types of neurotransmitters and neuropeptides onto primary afferents, the role of GABAergic interneurons remains crucial.

Based on the expression of transcription factors, different subtypes of spinal interneuron with distinct settling positions, neurotransmitter expression, and profiles of connectivity have been identified [70], among which a few have an inhibitory phenotype [9]. In particular, an adenovirus vector including a neuropeptide Y promoter has been recently used to discover, in the superficial dorsal horn, a subset of inhibitory GABAergic interneurons (AAV-NpyP) with the ability to prevent the conversion of touch-sensing signals into pain-like behavioral responses [71]. This class of interneuron receives mono- or polysynaptic excitatory inputs from touch-sensing fibers and uses GABA for transmitting inhibitory signals to lamina I neurons that project to the brain, thus avoiding abnormal excitation following innocuous mechanical stimulations (Fig. 2b). Dysfunctions of GABAergic transmission at the level of dorsal microcircuits impair the mechanisms of presynaptic inhibition, resulting in neuropathic pain states [72]. Neuropathic pain is one of the most frequent complications in paraplegics, with an incidence of 53% [73], and is often treated with GABAergic drugs [74, 75].

Indeed, the severity of neuropathic pain states following an experimental SCI [76] and other neurologic disturbances [77] is correlated to a reduced GABAergic tone, as the loss of GABAergic inhibitory interneurons in the superficial dorsal horn is verified by the reduction in GAD65/67 immunostaining. Thus, interventions for restoring the impaired production of GABA and GADs in the dorsal horns also alleviate pain states [77].

NKCC1 is crucial for the accumulation of Cl^-^ in DRG neurons, leading to depolarizing GABA responses on primary afferents. Different studies demonstrated a transient upregulation of NKCC1 at DRG neurons after nerve injury indicating that Cl^-^ efflux contributes to presynaptic inhibition and neuropathic pain induction [78–80]. Consequently, transgenic knockout mice lacking NKCC1 show impairments of presynaptic inhibition and significant alterations in locomotor and pain behaviors [53, 81]. Recently, disruption of NKCC1/KCC2 balance and chloride gradient below the injury site were found after spinal cord cervical contusion demonstrating the contribution of Cl^-^ homeostasis for spasticity and chronic pain [82]. Indeed, in a rat model of neuropathic pain, the use of the extrusion enhancer CLP257, a KCC2-selective analog that lowers Cl^-^ intracellular concentration, can alleviate hypersensitivity [83]. Hyperalgesia and allodynia were improved by using bumetanide for 2 weeks following sciatic nerve lesion, demonstrating the role of cation chloride co-transporter expression to modulate nociceptive pathways [84]. These data demonstrate that neuronal GABA neurotransmission is dependent on precise regulation of the level of intracellular chloride, which is determined by the coordinated activities of cation chloride co-transporters and could open new perspectives to prevent or alleviate neuropathic pain and functional recovery after SCI.

Collectively, these data show notably similar features between SCI and neuropathic pain, as they may both originate from alterations of presynaptic GABAergic mechanisms, which in turn broaden the potential translation of novel approaches to redress the tilted balance between excitation and inhibition in either neurological conditions.

## Glycine Is a Fast Inhibitory Transmitter in the Spinal Cord

In adult rats, GABAergic axon terminals represent only 20% of the inhibitory input converging onto lumbar motoneurons, while the remaining 80% are glycinergic [85].

Glycine is a fast inhibitory transmitter on spinal motoneurons [19, 86], and it might be co-released with GABA at certain synapses [87]*.* However, not all synaptic boutons on motoneurons have both inhibitory neurotransmitters, but rather a strong prevalence of glycine alone [88]*.* Postsynaptic GABA A and glycine receptors are often, albeit not necessarily, co-localized [89] and aggregated in clusters formed by the submembrane scaffolding protein gephyrin [90, 91].

The glycinergic system is relatively insensitive to spinal transection [92]. Indeed, both the density of glycine receptors on motoneurons and the kinetics of glycine-mediated currents remain unchanged [34]. In accordance with these observations, the concentration of glycine, as determined by HPLC on spinal cord homogenates (2–12 h after spinal cord contusion), is preserved [93]. Only much later (3 weeks from transection), the expression of glycine receptors is temporarily decreased with subsequent recovery and re-emergence of physiological reflexes [94]. After complete spinal transection, the comparatively well-preserved glycinergic system at segmental level below the lesion may represent one significant component for neurorehabilitation protocols [92].

Since the main focus of the present review manuscript is the dysfunction of GABAergic mechanisms in damaged spinal networks, we refer the reader to previous work to examine the role of glycine after SCI [34, 92, 94–97].

## Early Peak of GABA Immediately after SCI

Mechanical impact to the spinal cord massively increases the extracellular concentration of several neurotransmitters including GABA. Experimentally, a strong increase of GABA at the lesion site has been observed shortly after an SCI in vivo [42] following the very early rise in glutamate concentration (Fig. 3c). The increased extracellular concentration of GABA rapidly declines following SCI and later recovers to the pre-trauma levels [42, 93, 98]. The peak of GABA after SCI originates from not only the destruction of the membrane of GABAergic and glia cells but also the synaptic release at the site of injury [99] facilitated by spreading depolarization along the injured tissue [100]. The contribution of circulating GABA leaking through the impaired blood-spinal barrier is probably a minor one as GABA concentrations in the plasma [101, 102] are far below the ones found at the lesion site. Nevertheless, there might be enough GABA to activate highly sensitive extra-synaptic GABA receptors such as the ones incorporating the δ subunit [40]. An additional contribution to the peak in extracellular GABA immediately after SCI comes from the reversed function of membrane GABA transporters that depend on Na^+^ concentrations. In both neurons and glia, physiological reuptake of GABA is coupled to Na^+^ and Cl^-^ inflow into the cell [103]. The increased concentration of intracellular Na^+^ (and Cl^-^) caused by spreading depolarization following an acute injury reverts the transport systems to extrude GABA [104]. At the same time, downregulation of the vesicular GABA transporter caused by SCI [105] increases the amount of cytosolic GABA available for extrusion.

The peak of GABA corresponds to the onset of a transient depression of spinal reflexes below the level of injury named spinal shock [106] typically present after severe spinal contusions in rats [107], although rarely found after surgical transection of the cord [108]. We, therefore, propose a role for GABA in spinal shock alongside a similar role for glycine [96].

## Fast Synaptic GABAergic Transmission Is Early Affected by Spinal Cord Injury

The excitation/inhibition balance ensures physiological motor responses executed by healthy spinal cords and may be directly altered by SCI. Future studies are required to clearly identify the components of the locomotor systems primarily altered after SCI and their impact on the excitation/inhibition balance. In broad terms, changes in excitation/inhibition balance might originate from an alteration in cellular mechanisms and/or disruption and rewiring of local networks. Hence, in response to spinal damage, GABAergic cells show particular vulnerability, as their number decreases [109]. One reason for their vulnerability might be their location because important members of the spinal GABAergic population are commissural interneurons, which cross the midline and project ventrally, thus offering a long section liable to injury [110]. Furthermore, the ventral region is vulnerable to SCI because of its dense vascularization prone to produce large hemorrhage and neuronal loss [111]. In addition, in the acute phase of SCI, complex neurodegenerative events develop to generate a secondary injury that amplifies and spreads damage to the neighboring tissue [112]. Our former studies have provided a comparative description of the different neuronal cell types with particular vulnerability to injury [25, 113, 114]. In the early phases of experimental SCI, significant reduction in GABAergic GAD65 expression occurs at the injury site [115].

One important contributor to secondary injury is the over-activation of glutamate receptors, leading to a massive influx of calcium ions into spinal cells and contributing to the release of free radicals from mitochondria, such as reactive oxygen and nitrogen species, in turn triggering intracellular toxic cascades (excitotoxicity; [25, 113, 116–118]).

The oxidative stress occurring during secondary damage is one important cause for the impairment in GABAergic neurotransmission, because reactive oxygen species increase synaptic release of GABA [119, 120] that desensitizes GABA ARs [121]. Reactive oxygen species also alter the function of GABA A receptor-gated Cl^-^ channels due to a reduced driving force for Cl^-^ because of failure of its transport [122]. In addition, free radicals alter the binding characteristics of GABA, possibly by affecting redox-sensitive receptor sites or via peroxidation of membrane lipids surrounding the receptor [122].

GABAergic descending inputs that control motoneuron excitability are also damaged by SCI contributing to functional motor deficits and other disabling consequences. In the majority of people with chronic SCI, paralyzed muscles are often accompanied by involuntary contractions (spasticity), increased resistance to passive stretch (muscle hypertonia), and exaggerated motor responses to light peripheral stimulation (hyperreflexia; [123]). Indeed, despite the reduced excitability of axons at the periphery [124], a brief sensory stimulation (< 20 ms) evokes a prolonged depolarization (~ 1 s) of single motor units apparently without efficient synaptic inhibition. Conversely, the same light afferent stimulus applied to neurologically intact subjects generates a sustained depolarization interposed by an inhibitory phase [125]. The increased amplitude that characterizes motor responses after SCI and the lack of inhibitory contributions have been associated with multiple neuronal mechanisms at both cell and network levels. While the increased excitation should be, at least in part, attributed to the activation of Na^+^ and Ca^2+^ persistent inward currents (PICs) in motoneurons [126–130], a pivotal role in reduced inhibition has been ascribed to depression in GABAergic transmission [92, 131]. Indeed, at pre-synaptic level, despite the increased size of GABAergic synapses, the lower number of vesicles in the active zone [132] determines less neurotransmitter available for release. At the same time, an SCI also produces aberrant hyper-connectivity among GABAergic interneurons, with the formation of new axo-axonic synapses [132] that, along with changes in Cl^-^ transporter isoforms, might contribute to the disinhibition reported after SCI [133].

Noteworthily, dysregulation of the balance between excitation and inhibition may also result from changes in other components of the spinal network after injury. For instance, aberrant sprouting of primary afferents or expansion of interneuronal receptive and projective fields after SCI may augment the excitatory drive to spinal networks [134]. On the other hand, inhibition is affected by the interruption of serotoninergic descending tracts, which modulate inhibitory interneurons, like Renshaw cells [135, 136]. Moreover, Renshaw cell recurrent circuitry might become disconnected from motoneurons [137] suppressing their excitatory drive to Renshaw cells, in turn reducing the GABAergic inhibitory feedback. Also, changes in long-term gene expression, such as upregulation and phosphorylation of several signaling proteins in spinal ventral horns, have been linked to early and long-term changes in spinal excitability, leading to spasticity states after spinal trauma [138].

Furthermore, circuit reorganization after spinal cord injury occurs also at the supraspinal level. The strength of brainstem reflexes is enhanced as a result of increased excitability and reduced GABA-mediated inhibition in the brainstem circuits that project to spinal interneurons [139].

Table 1 shows interventions aimed at normalizing the altered excitability after injury from multiple experimental settings. Pharmacological manipulations, transplants of different cell lineages, and activity-dependent protocols have been applied in the acute and chronic phases of SCI to exploit GABA-related mechanisms and rescue homeostasis between excitation and inhibition.

**Table 1:** GABAergic mechanisms targeted to rescue altered inhibition

Intervention		Model	GABAergic mechanisms	Main outcome	Reference
Physical exercise	• Treadmill running	• Partial sciatic nerve (PSL) ligation in adult C57BL/6 J mice	• Restoration GABAergic interneuron numbers • Upregulation of GAD65/67 immunoreactivities	• Alleviates allodynia and heat hyperalgesia • Positive correlation between GABA levels and the thresholds of von Frey or plantar tests	Kami et al. (2016) [77]
Physical exercise & Pharmacology	• Cycling exercise • Bumetanide (NKCC1 antagonist) • DIOA([(dihydroindenyl)oxy]alkanoic acid; KCC2 antagonist)	• SCI complete transection in adult female Sprague Dawley rats	• Increase in KCC2 levels and decrease in NKCC1 expression levels • Blockage of NKCC1 impacts on reflex recovery • Apparent modulation of KCC2, but not NKCC1, by BDNF	• Exercise contributes to functional recovery by restoring chloride homeostasis	Côté et al. (2014) [67]
Pharmacology	• CLP290 (KCC2 agonist) • Bumetanide (NKCC1 inhibitor) • 8-OH-DPAT (5HT1A/7 agonist) • Quipazine (5HT2A/C agonist) • CP101606 (NMDA receptor antagonist) • Baclofen • L838414 (GABA A-positive allosteric modulator)	• SCI bilateral hemisection in adult mice	• Increase in KCC2 function	• Restores inhibition in the injured spinal cord, leading to functional recovery	Chen et al. (2018) [140]
	• CLP257 • CLP290	• NG-108 cell line and HEK293-cl cells • Horizontal spinal dorsal horn slices obtained from animals with peripheral nerve injury (PNI) • PNI in adult male Sprague-Dawley rats	• CLP257 and CLP290 enhance Cl^−^ extrusion	• CLP257 has antinociceptive properties in PNI animals	Gagnon et al. (2013) [83]
	• Intrathecal administration of brain-derived neurotrophic factor (BDNF) and of BDNF sequestering agent, TrkB-IgG	• SCI transection in adult male Sprague-Dawley rats	• Increase in KCC2 expression post-SCI by BDNF	• BDNF plays an antinociceptive role	Huang et al. (2017) [141]
	• Activation of 5-HT2A receptors with TCB-2	• SCI hemisection in adult female Wistar rats • Peroneal and tibial nerve injury by ligation and transection • Injection of TCB-2 and intrathecal DIOA injection	• Increase in membrane KCC2 expression	• Restores motoneuronal inhibition, and reduces SCI-induced spasticity, mechanical and thermal hyperalgesia • Nerve injury-induced neuropathic pain was not attenuated by TCB-2	Sánchez-Brualla et al. (2018) [142]
	• Midazolam (allosteric GABAA modulator) • THIP (GABA agonist) • Bicuculline • Gabazine (antagonist of GABA ARs) • Strychnine • L-Alanine	• Mouse organotypic spinal slice cultures, excitotoxicity induced by kainate	• Increase in GABA receptor activity through pharmacological GABA agonism	• Decreases excitotoxic death in spinal networks in vitro	Mazzone and Nistri (2019) [143]
	• TGN-20 (AQP4 inhibitor) and bumetanide	• SCI contusion rats	• Upregulation of AQP4 mRNA and reduction of NKCC1 expression	• Reduces SCI edema and tissue destruction	Yan et al. (2018) [68]
	• Anodal trans-spinal direct current stimulation and bumetanide	• SCI contusion in CD-1 mice	• Upregulation of NKCC1	• Reduces spasticity and increases muscle tone	Mekhael et al. (2019) [69]
Transplantation	• Transplantation of MGE-like cells derived from human embryonic stem cells (hESC-MGEs)	• SCI moderate contusion in B6.CB17- Prkdcscid/SzJ transgenic mouse	• Migration and differentiation into GABAergic neurons subtypes	• Transplanted cells functionally integrate into host’s spinal cord • Attenuate mechanical allodynia of hind paws • Sustained motor recovery	Fandel et al. (2016) [144]
	• Transplantation of embryonic precursors of GABAergic neurons from medial ganglionic eminence (MGE)	• Peripheral nerve injury models of neuropathic pain in adult mouse	• Differentiation into GABAergic neurons	• Transplanted cells functionally integrate into host’s dorsal horn circuits	Llewellyn-Smith et al. (2017) [145]
	• Transplantation of fetal neural stem cells (fNSC) extracted from the telencephalic vesicles (TV) and the ventral medulla (VM)	• SCI contusion in adult Wistar rats	• Differentiation into GABAergic neurons • Greater proportion of GABAergic cells from the TV group compared to the VM group	• Improves from thermal hyperalgesia • Ameliorates mechanical allodynia	Batista et al. (2019) [146]
	• Transplantation of differentiated human induced pluripotent stem cell-derived GABAergic (iGABAergic) neurons	• Peroneal and tibial nerve injury by ligation and transection in adult mice	• Differentiation into GABAergic neurons. • VGAT and GAD65/67 expression	• Transplanted cells functionally integrate into host’s dorsal horn active inhibitory circuits • Reduces tactile allodynia	Manion et al. (2020) [147]
	• Transplantation of GABAergic neural progenitor cell and intensive locomotor training (ILT)	• SCI compression in adult male Sprague Dawley rats	• Upregulation of KCC2	• Reduces mechanical allodynia and thermal hyperalgesia • Reduces pro-inflammatory markers	Dugan et al. (2020) [148]
Genetic manipulation	• NKCC1 gene ablation in DRGs • Bumetanide	• NKCC1 knockout mice, deletion of exon 9 of the gene	• Absence of Cl- accumulation in DRGs • Absence of GABA depolarizing responses	• Alters nociception and motor coordination	Sung et al. (2000) [53]

Despite the plethora of experimental approaches, restoring physiological spinal inhibition in the clinic remains a timely and demanding challenge that requires further studies. Indeed, potentiating the GABAergic system, when not carefully timed, might even hinder activity-based rehabilitation and electrical neuromodulation protocols for motor recovery, by depressing synaptic transmission [149] and reducing excitability of locomotor spinal circuits [150].

## Pharmacological Neuroprotection by GABA Modulation after Experimental Lesion

Several GABAergic mechanisms targeted at restoring functional homeostasis and rescuing neuronal loss after injury have been explored with different experimental models (Table 1). For their part, reduced preparations from neonatal rodents suggest that a large rise in extracellular glutamate is responsible for the excitotoxicity arising early after SCI (Fig. 3c). In this model, excitotoxicity is produced by transient application of the powerful glutamate analog kainate [151]. While glutamate excitotoxicity can be attenuated with agents that decrease its release [152–156], a distinct approach is to boost inhibition to render spinal neurons less excitable. Thus, neuroprotection by general anesthetics like methoxyflurane and propofol indicates that this process effectively counteracts excitotoxicity [157–159] albeit through distinct molecular mechanisms. In fact, while methoxyflurane primarily acts by hyperpolarizing motoneurons via opening a voltage-independent K^+^ channel [159], propofol enhances GABA ARs activity by binding to a discrete allosteric site [158]. The implication of these results is that neuronal inhibition, regardless of its effector mechanisms, is an important factor to contrast excitotoxicity. Nevertheless, using general anesthetics as a neuroprotective drug is complex and prompts the search for alternative approaches. In line with this strategy, more direct investigation into the effects of GABA receptor agonists and antagonists on experimental spinal damage has shown that modulation of extrasynaptic GABA ARs could prevent excitotoxic death of spinal organotypic cultures [143]. In particular, the allosteric GABA A modulator midazolam and the GABA agonist 4,5,6,7-tetrahydroisoxazolo [5,4-c] pyridin-3-ol (THIP; preferentially acting on extrasynaptic receptors) are powerfully effective [143]. In addition, the GABA AR antagonist bicuculline prevents the neuroprotective effect of propofol via GABA AR function, suggesting the importance of GABA receptor activity in modulating excitotoxicity [157]. Endogenous neurosteroids can also induce neuroprotection by upregulating GAD67 enzyme level [160] or GABA AR function [161]. Thus, even if transient changes in GABAergic synaptic transmission after SCI might not be immediately translated into neuroprotection, other GABAergic targets are available to perform this role. Interestingly, cultured motoneurons show that the excitotoxic action of glutamate is limited by direct application of GABA agonists [162, 163].

The neuroprotective role of GABA as well as the activation of different GABA receptors following insults to the CNS [15] may represent potential targets to limit damage and develop innovative and selective therapeutical approaches.

However, side effects of current pharmacological therapy for other neurological disturbances, as epilepsy, suggest potential risks from potentiating GABAergic mechanisms [164]. Likewise, the use of the anticonvulsant baclofen determines muscle weakness and sedative effects [165], along with a baclofen-withdrawal syndrome, with a psychotic status when the drug is abruptly discontinued [166]. However, since GABA BRs are less prone to receptor desensitization, the abovementioned adverse effects are likely to be more pronounced than interventions targeted to GABA ARs.

## Neurons and Astrocytes May Counteract Excitotoxicity via GABAergic Mechanisms

One key element to modulate synaptic transmission and neuronal network activity seems to be the presence of astrocytes and the type of neuron involved [167]. It is now widely accepted that astrocytes can modulate neuronal activity through the tripartite synapse [168]. Thus, cells immunoreactive to S100β (a cytoplasmic calcium-binding protein mainly expressed by glia), may take part in tissue protection and repair, as well as they are useful biomarkers for brain or spinal cord injury [169]. These cells are the most abundant astrocyte cell type in the ventral horn area and less abundant in the dorsal horn [170]. The differential distribution of glial cells within the spinal cord regions might be an important factor in considering the high vulnerability of neurons to excitotoxicity [25, 113, 114]. Accumulating evidence demonstrates the role of astrocytes in GABA synthesis and release, as well as in the activation of GABA receptors on neighboring neurons [60]. During synaptic transmission, GABA release triggers astrocytic release of calcium from the endoplasmic reticulum via the inositol 1, 4, 5-trisphosphate pathway [171]. As pointed out by Christensen and collaborators [172], in the dorsal horn of adult turtle, astrocytes coordinate calcium-mediated excitation and tonic inhibition by GABA ARs to induce phasic release of GABA. Finally, lampreys show spontaneous functional recovery and neuroprotection after complete SCI that depends on astrocytes properties related to GABA accumulation and neurotransmitter uptake [173].

Although promising for the design of novel interventions to rescue cellular loss after spinal damage, these results must be considered with caution and must be supported by compelling new studies to validate any translation to clinical use. Potential limitations can originate when interpreting results coming from different species, genders, age, phases of lesion, and injury protocols (Table 1). In fact, the distribution of GABA ARs and their binding properties might vary among different strains [174], while also circulating sex hormones affect the sensitivity of GABA ARs to the allosteric endogenous modulator allopregnanolone in females [175]. Moreover, mechanical properties of the spinal cord change with size, making it hard to compare the severity of experimental injuries among studies of animals at different developmental stages [176].

## Prolonged Dysfunction of Fast GABAergic Transmission after SCI

After spinal cord transection, the number of GABA ARs increases in fast flexor motoneuronal pools and synaptic clustering augments as a consequence of subunit overexpression. This latter feature is reversed to control after step training and aids functional recovery [177]. Furthermore, long-term changes in protein and mRNA levels of GAD67 (but not GAD65) have been found after a chronic transection, possibly leading to increased GABA production in spinal neurons below the site of injury [29]. Interestingly, GAD67 is the predominant form in ventral horn neurons around motoneuronal pools [178] and the recovery of locomotor functions in SCI rats corresponds to a return of GAD67 toward baseline levels [179].

Enhancement in motoneuron excitability stems from their dysregulation of intracellular Cl^-^ caused by the spinal lesion itself [180]. In lumbar motoneurons, thoracic SCI reduces the expression of KCC2 which co-transports potassium and Cl^-^ outside the cell [181]. The switch of GABA A from inhibition to excitation contributes to the spasticity of hind limbs [182]. In fact, upregulation of KCC2 after transection restores some locomotor activity in the mouse [140].

The interaction between excitation and inhibition at chronic stages of SCI remains an incompletely understood process as much as the relative weight of GABA and glycine mediated transmission. In fact, although glycine receptor operation is also sensitive to intracellular Cl^-^ [183, 184], the kinetics of glycinergic currents are not affected after spinal transection [34] and the administration of glycine continues to produce inhibitory effects and limit spasticity after SCI [95]. Pharmacological block of both GABA A and glycine receptors prolongs spasms in chronically transected animals, confirming that a degree of fast inhibition remains efficacious even after lesion [95, 185]. In keeping with these observations, optogenetic activation of spinal inhibitory interneurons silences spasms evoked by electrical afferent stimulation [185]. Conversely, Edgerton and Roy [186] have proposed low doses of pharmacological blockers of Cl^-^-mediated inhibition for recovery of gait in injured animals. Antagonism of inhibitory transmission has been claimed to facilitate locomotion by limiting excessive inhibition following SCI [97, 187, 188].

In sum, after SCI, the excitability of spinal networks at rest is changed at distinct nodes of the pre-motor neuronal circuitry by the appearance of complex contributions with a very fine balance among them. On the one hand, GABA-mediated depolarizing signals result from the reversed Cl^-^ gradient [182, 189]. On the other hand, supplementary GABA-mediated inhibitory input arises from upregulation of GABA synthesis [178], overexpression of GABA AR subunits [34, 177], and a greater activation of inhibitory interneurons [185]. Ultimately, whether synaptically released GABA can either inhibit or facilitate excitatory inputs depends on the time course of the event and its membrane topography on the postsynaptic neuron [39]. Hence, the longer lasting the effect of GABA is, the higher is the likelihood of inducing neuronal excitation.

## Factors Regulating the Excitability of Motoneurons after SCI

First, chronic changes in motoneuronal excitability after human SCI depend on how close these cells are to the site of spinal injury. Namely, while perilesional motoneurons are hypo-excitable, those farther from the lesion epicenter show increased excitability [190]. In line with this finding, in subjects with incomplete SCI, corticospinal pathways evoke aberrantly high facilitation of motor output distant from the epicenter of the lesion. Conversely, no change is reported at the level of injury and nearby segments [191]. Animal experiments indicate that sustained depolarization of sacral motoneurons below the lesion [192] is accompanied by hypertonia, hyperreflexia, and clonus [193, 194]. Other studies have demonstrated aberrant membrane properties of lumbar motoneurons underlying hind limb spasticity after thoracic spinal lesions in rodents although direct evidence for the excitability of motoneurons close to the contusion site is still missing [181, 189]. While motoneuron properties (essential to support motoneuron firing) slowly recover to their preinjury state, their corresponding receptive fields remain broad so that sensory input to even a small area of the limb can trigger widespread excitation capable of generating whole-limb spasms [195]. Further studies are eagerly awaited to explore whether different states of excitability of motoneurons proximal and distal to an SCI are related to the early transient changes in extracellular GABA concentrations at the epicenter of injury. Potentially, these findings might bring novel pharmacological interventions to acutely modulate GABAergic transmission below the lesion [196] with the timely goal of preventing the onset of spasticity in addition to the widely-used administration of the GABA BR agonist baclofen [197]. In particular, an important issue is whether activation of spinal GABA ARs may be able to counteract the upregulation of the persistent sodium current of motoneurons typically observed after lesion [198]. This conductance is considered to be the target for neuromodulation, a phenomenon in which GABA is expected to play a role [199]. PICs which comprise sodium as well as calcium conductances [126–130, 200] contribute to the nonlinearity between the level of network excitation and motor output [201]. As spinal neurons possess strong plasticity during recovery after SCI [202], GABA AR currents display more powerful control over PIC activation than glycinergic currents, an effect attributable to their slower kinetics [196]. Additionally, extrasynaptic GABA ARs (with their high sensitivity to even low GABA concentrations) may represent a further mechanism to downplay neuronal excitability even when synaptic transmission has failed after SCI. Nevertheless, the functional outcome of modulation by GABA receptor activity may also depend on the shifting balance between hyperpolarizing and depolarizing action of GABA due to post lesional changes in chloride transmembrane gradient [140, 180–182, 189] and their timing as discussed earlier.

In conclusion, restoration of locomotor network activity after injury depends on the correct interplay between excitation and inhibition and recovery of the fine balance between synaptic and non-synaptic GABA AR activity. These goals are eminently suitable for pharmacological investigations.

We suggest that this is a complementary strategy to concur with the use of new materials and cell transplants to a successful repair or reconfiguration of damaged locomotor networks that need a suitable functional milieu to reestablish their correct operation.

## Abbreviations

5HT: Serotonin
CPG: Central pattern generator
DRG: Dorsal root ganglion
GABA: Gamma-aminobutyric acid
GAD: Glutamic acid decarboxylase
GFP: Glial filament protein
L: Lumbar
MN: Motoneuron
NeuN: Neuronal nuclear protein
NMDA: *N*-methyl-d-aspartate
NS: Nociceptive-specific projection neuron
PICs: Persistent inward currents
ROIs: Regions of interest
SCI: Spinal cord injury
SD: standard deviation
THIP: 4,5,6,7-Tetrahydroisoxazolo [5,4-c] pyridin-3-ol
VGAT: vesicular GABA transporter
VRs: ventral roots
